# Evaluating hierarchical machine learning approaches to classify biological databases

**DOI:** 10.1093/bib/bbac216

**Published:** 2022-06-21

**Authors:** Pâmela M Rezende, Joicymara S Xavier, David B Ascher, Gabriel R Fernandes, Douglas E V Pires

**Affiliations:** Universidade Federal de Minas Gerais; Instituto René Rachou, Fundação Oswaldo Cruz; Stilingue Inteligência Artificial; Universidade Federal de Minas Gerais; Instituto René Rachou, Fundação Oswaldo Cruz; Institute of Agricultural Sciences, Universidade Federal dos Vales do Jequitinhonha e Mucuri; School of Chemistry and Molecular Biosciences, University of Queensland; Systems and Computational Biology, Bio 21 Institute, University of Melbourne; Computational Biology and Clinical Informatics, Baker Heart and Diabetes Institute; Instituto René Rachou, Fundação Oswaldo Cruz; Systems and Computational Biology, Bio 21 Institute, University of Melbourne; Computational Biology and Clinical Informatics, Baker Heart and Diabetes Institute; School of Computing and Information Systems, University of Melbourne

**Keywords:** class hierarchy, hierarchical classification, biological database, protein function prediction, protein structural classification

## Abstract

The rate of biological data generation has increased dramatically in recent years, which has driven the importance of databases as a resource to guide innovation and the generation of biological insights. Given the complexity and scale of these databases, automatic data classification is often required. Biological data sets are often hierarchical in nature, with varying degrees of complexity, imposing different challenges to train, test and validate accurate and generalizable classification models. While some approaches to classify hierarchical data have been proposed, no guidelines regarding their utility, applicability and limitations have been explored or implemented. These include ‘Local’ approaches considering the hierarchy, building models per level or node, and ‘Global’ hierarchical classification, using a flat classification approach. To fill this gap, here we have systematically contrasted the performance of ‘Local per Level’ and ‘Local per Node’ approaches with a ‘Global’ approach applied to two different hierarchical datasets: BioLip and CATH. The results show how different components of hierarchical data sets, such as variation coefficient and prediction by depth, can guide the choice of appropriate classification schemes. Finally, we provide guidelines to support this process when embarking on a hierarchical classification task, which will help optimize computational resources and predictive performance.

## Introduction

Biological databases play an important role in contemporary research, providing curated and annotated data sets for use across many fields, including medicine, chemistry and biotechnology. Biological data are usually deposited by researchers, collected from literature or derived from computational analysis. Since this information retrieval involves human intervention, it is prone to errors and, considering the amount of biological data routinely collected [1], automatic data classification is often required to leverage the wealth of information within these repositories.

Many biological data sets are hierarchical in nature, which means they have classes (or labels) that can be further divided into other classes, such as organism taxonomy [2, 3], structural domains of proteins [4–7], metabolic pathways [8, 9], enzyme classifications [9, 10], among others. In contrast to flat classification, where classes are considered unrelated and independent, hierarchical classification associate labels to different classification levels [11]. These hierarchies become a challenge to traditional classification algorithms as they are, in general, not well equipped to address large-scale problems involving hundreds of thousands of hierarchically related classes, which is often the case for real biological datasets [11]. For example, a taxonomic representation of the archaea domain possesses around 32 000 classes per level, a degree of complexity that has led to common classification inconsistencies [12, 13].

Previous efforts have shown that classifiers tailored to this type of complex hierarchical data improve information retrieval effectively [11, 14, 15]. Silla and Freitas, for instance, describe the main challenges in hierarchical classification tasks, including class unbalance and a high number of classes, prediction by depth, and the classification in deeper levels [15]. Over the years, many hierarchical classification methods have been proposed, including new evaluation metrics [11] and deep learning approaches [16–17]. These have been, however, mainly applied to text classification problems [18], with little work devoted to tackling the challenges of hierarchical classification on biological databases. Furthermore, within the field of Bioinformatics, hierarchical frameworks have been used for specific domains of application [19–23]; however, they mainly comprise taxonomic databases, which present significant limitations, particularly due to their level of curation and data quality [12, 13, 24–27]. Little, therefore, has been done to comprehensively assess the utility, applicability and limitations of different hierarchical classification approaches applied to different biological databases [28]. In this work, we evaluated the approaches proposed by Silla and Freitas [18] and applied them to different biological databases to investigate their pros and cons and establish general guidelines of practice.

Two curated databases presenting different hierarchical problems were selected from distinct areas to undergo automatic classification: CATH [29] and BioLip [30]. CATH is a database that maps evolutionary relationships on protein domains, which are classified into four levels: class, architecture, topology and homologous superfamily [31]. The main classification challenges related to CATH include a high number of classes at deep levels, full depth labeling and the highly unbalanced nature of classes. BioLip, on the other hand, is a database of ligand–protein binding interaction data [30]. From this database, we extracted enzyme classification as a proxy for protein catalytic function, expressed as an Enzyme Commission number. BioLip, contrary to CATH, does not accept full depth labeling but presents highly unbalanced classes.

Here, we assessed and compared, for the first time, the performance of three hierarchical classification approaches on three data sets [Global, Local per Node (Node) and Local per Level (Level)] and provide guidelines to choose appropriate strategies to classify hierarchical datasets considering their main characteristics.

## Hierarchical classification

In traditional or flat classification, a model is trained to assign each object to a single class belonging to a finite number of classes. When the object is associated with different classification levels, however, there is a specialization of this task, named hierarchical classification.

Hierarchical classification can be organized as either a tree or a directed acyclic graph (DAG) topology. In a tree topology, each child-class is associated with a single parent-class—or ancestor—(Supplementary Figure S1A available online at http://bib.oxfordjournals.org/), whereas in a DAG topology each child-class can be associated with one or more parent-classes (Supplementary Figure S1B available online at http://bib.oxfordjournals.org/) [11]. The main difference between the topologies is in the classification result: while in a tree there is a single path to classify each leaf node, in a DAG there may exist more than one.

Hierarchical classification can be categorized based on three main characteristics [18]:

(i) Hierarchy type, in which the classes are organized (Tree or DAG).
(ii) Single or multi-label classification (i.e. allowing for data points to follow multiple classification paths).
(iii) Based on data labeling depth, that is, either all instances have labels until the leaf nodes, which represent the deepest levels in a hierarchy, or partial depth labeling.

### Challenges in hierarchical classification

Considering $X$ as the spaces of instances, a hierarchical classification problem consists of finding a function (classifier) $f$to map each instance ${x}_i\in X$ to a set of classes ${C}_i\in C$, with $C$ being the set of classes in the problem. The function $f$ must respect the constraints of hierarchy and optimize a quality criterion [32]. As constraints of the hierarchy, when a class is predicted, all its superclasses should also be automatically predicted.

Hierarchical classification problems are formally defined as tuples ($\gamma, \psi, \phi )$, where $\gamma$ specifies the topology (Tree or DAG), $\psi$ describes whether the instances are classified into multiple paths of labels or into a single path of labels and $\phi$ dictates if the classification can stop at an internal node of the hierarchy (non-mandatory leaf node or partial depth labeling (PD)), or if it must continue until a leaf node is reached (mandatory leaf node or full-depth labeling (FD)) [18, 32].

**Figure 1:**
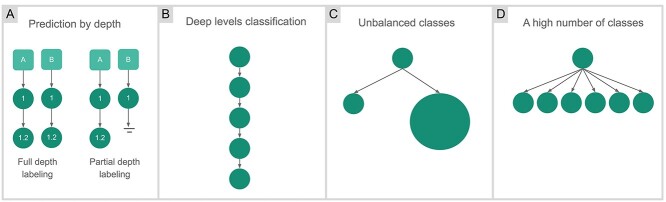
Representation of challenges faced in hierarchical data classification. (**A**) Prediction by depth: Describes the label depth of the data instances. Full-depth labeling indicates that every instance is labeled with classes at all levels, from the first level to the leaf level. Partial depth labeling indicates that at least one instance has a partial depth of labeling, i.e. the value of the class label at some level is unknown. (**B**) Deep levels classification: The complexity of classifying is affected by the number of levels in the topology. (**C**) Unbalanced classes: There is an unequal distribution of classes in the dataset, which could penalize lower classes in the classification process. (**D**) A high number of classes: An expressive number of classes, mainly in the last level, affects the complexity of the model.

As the number of levels in a hierarchy increases, the complexity and effort required to achieve a satisfactory prediction increase. Prediction by depth can be used through two strategies: full or partial depth labeling (Figure 1A). Full-depth labeling is used when every node should be classified in all hierarchy levels, from root to leaf nodes. The disadvantage of full labeling is that data are assigned a class regardless of prediction confidence. In partial depth labeling, in contrast, the prediction task is interrupted when the confidence is low, ensuring classification reliability. Besides the problems related to the topology, often observed in biological datasets, two common challenges may generate bias in classification models: unbalanced classes (a well-known challenge for machine learning models, which tend to privilege majority classes) and large numbers of classes (which reduce class boundaries, making the learning process more difficult).

In summary, the four main challenges in hierarchical classification are as follows (Figure 1):

- Prediction by depth: The instances should be classified until the last hierarchy level or until prediction accuracy is sufficient.
- Deep levels of classification*:* The deeper a classification level is, the more difficult it is to achieve good prediction accuracy.
- Unbalanced classes: A large difference in the number of instances between different classes.
- High number of classes: A predictive model for a large number of classes needs to be trained.

### Hierarchical classification approaches

Two main approaches have been used to deal with hierarchical classification problems: local and global techniques.

**Figure 2:**
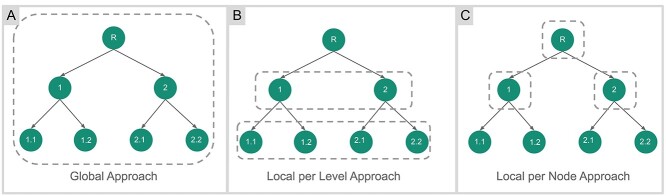
Hierarchical classification approaches. (**A**) Global Approach: Considers the entire class hierarchy at once. (**B**) Local per Level Approach: Consists of training one multiclass classifier for each level of the class hierarchy. (**C**) Local per Node Approach: Consists of training a multi-class classifier for each parent node in the class hierarchy. (Adapted from Silla and Freitas [18]).

Global classification looks at classification paths as a single label. Data hierarchy is disregarded and the classifier works as a flat classifier, i.e., a single predictive model is generated for all hierarchy levels. In contrast, Local approaches, which are divided into Node and Level-based, consider label hierarchy. Figure 2 shows the differences between approaches, where the dashed lines are the generated predictive models.

In the Level approach, a classifier is developed at each level of the hierarchy considering all nodes from each level as a class. Considering the example in Figure 2B, two classifiers would be trained, one for each class level to predict one or more classes at its corresponding class level.

The Node classification approach consists of developing a multi-class classifier for each parent node from the class hierarchy. Usually, the Node classification approach follows a mandatory leaf node prediction, since this approach associates a multi-class classifier to each internal node of the hierarchy. Therefore, each node learns to differentiate between its subclasses.

In terms of number of models, the label pool for each classifier in the Node approach will be its children nodes. In this approach, we have fewer classes per model in comparison with the Global approach; however, it produces considerably more models (with less information per model available for training).

In the Level approach, a classifier is developed for each level of the hierarchy considering all nodes from each level as a class. The Level approach produces fewer models than the Node approach. However, given the increase in the number of classes due to handling the entire hierarchy level, the Level approach generates more complex models [18].

**Figure 3:**
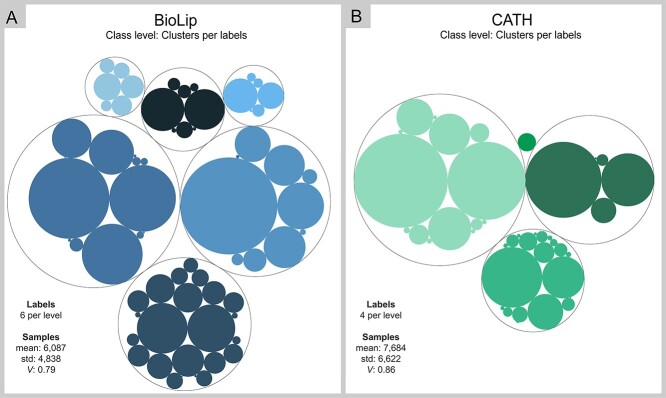
Distribution of entries in each class of BioLip (**A**) and CATH (**B**) from a top-down perspective. The division of clusters means the first level of classes in each one and the size presents the number of samples for each class.

## Methods

### Data set selection

The evaluation described in this study focuses on two hierarchical databases (CATH and BioLip) selected for a number of reasons. Firstly, their structure represents the challenges faced by hierarchical classification (depicted in Figure 1). CATH presents two challenges for classification: a high number of classes, and unbalanced classes, with a full-depth labeling scheme, while BioLip presents partial depth labeling and unbalanced classes as its main challenges. Additionally, these resources were chosen because of their popularity and their high level of curation and data quality, which limit confounders in the analysis and minimize classification errors. We assessed alternative publicly available biological hierarchical databases (Supplementary Table S3 available online at http://bib.oxfordjournals.org/) and identified potential alternatives (e.g. Silva [3] and KEGG [8] databases). However, they did not present the same level of curation as the other resources, nor did they belong to domains of applications already covered by CATH and BioLip.

### Algorithm selection

To prioritize the learning algorithms used in the experiments, we performed model selection in CATH, BioLip and Silva, using 10-fold cross-validation for 7 different algorithms (Supplementary Table S4 available online at http://bib.oxfordjournals.org/). Random Forest, Decision Tree and Extra trees had the best performances.

### Exploratory analyses

Our analysis used hierarchical classification approaches defined in the literature to evaluate their utility and applicability [18]. The classification algorithms used were implemented in Python, using the Scikit-learn library [33]. The configuration of machines used to perform the experiments is described in Supplementary Figure S2 available online at http://bib.oxfordjournals.org/.

Freely available releases of CATH and BioLip were downloaded (Supplementary Figure S3 available online at http://bib.oxfordjournals.org/). The hierarchical classification problem for CATH is described as $\gamma =\mathrm{tree},\psi =\mathrm{SPL},\phi =\mathrm{FD}$, and in BioLip, it is described as $\gamma =\mathrm{tree},\psi =\mathrm{SPL},\phi =\mathrm{PD}$. Both databases contain four levels of classification, and at each level, we can observe an unbalanced class representation. From a top-down perspective in Figure 3, it is possible to observe the distribution of classes in both data sets. The division of clusters shows the first and second levels of the hierarchy, highlighting the highly unbalanced nature and the variable number of classes at different levels.

Regarding topology, as we go down the tree level, both present an exponential growth of the number of classes or labels (Table 1 and Supplementary File 1 available online at http://bib.oxfordjournals.org/). In the last level, there is an average of 9 classes per node for CATH and 23 for BioLip, with a mean representation of 46 and 177 samples per class, respectively. The disparity between the mean and the standard deviation (SD) of the data indicates a high dispersion in the dataset. The ratio between these measures, called Variation coefficient (*V*), shows the degree of variation of the samples at each level. *V* is largely used to measure data dispersion or to evaluate problems in experiment results [34–36]. *V* indicates how large within-group differences tend to be in comparison with their average. The threshold used to evaluate the dispersion of a set varies according to the domain. However, in terms of statistical distribution, the SD of an exponential distribution is equal to its mean, so its *V* is equal to 1. Distributions with $V<1$ are regarded as low variance, while those with $V>1$are considered high variance [37].

**Table 1:** Characterization of databases in terms of classes and samples per level

		1° Level		2° Level		3° Level		4° Level	
		CATH	BIOLIP	CATH	BIOLIP	CATH	BIOLIP	CATH	BIOLIP
Labels	Per Level	4	6	26	23	520	32	654	206
	Per Node	–	–	10	11	46	6	9	23
Samples	Mean	7684.25	6087.67	1182.19	1578.52	59.00	1121.69	46.00	177.31
	SD	6622.29	4838.16	2310.56	2351.38	291.00	2868.25	509.00	532.99
	*V*	0.86	0.79	1.95	1.49	4.93	2.56	11.07	3.01

Table 1 shows that, in the first level, CATH and BioLip have almost the same variation, which is lower than 1. In the second level, this value increases consistently, mainly in CATH. From the third level on, the differences between datasets become more prominent, with the SD for the last level of CATH being 11 times higher than its mean, as opposed to the SD in Biolip, which is three times higher. These characteristics reinforce the classification challenges we presented previously: a high number of classes for the same node, an unbalanced representation of these classes and the difficulty to classify nodes as the level becomes deeper.

Regarding the prediction by depth problem, in BioLip, a sample is not always annotated until the last level, allowing a partial depth labeling. Therefore, if the class of the last levels is unavailable or duplicated, the last classification is considered as a leaf.

### Feature engineering

We used amino acid composition descriptors from *iFeature* [38] to represent proteins in Biolip [38] as these have been broadly used in previous work modeling information in this database [39–41]. Graph-based signatures were also used to represent protein structures in CATH, as they have been previously used to model protein structure and function [42–45], predict effects of mutation [46–52] and model CATH hierarchy [7]. Here, we chose well-known and validated descriptors for each database, since evaluating optimal descriptor sets was out of the scope of the present work.

To simplify the predictive models and reduce computational time requirements, we performed a feature selection using Shapley values [53], which explain the feature contribution in all combinations of possible features from a supervised model. We evaluated the model using Matthew’s correlation coefficient (MCC) and Recall. For BioLip, the best 60 features ranked by Shapley value were selected. We observed no change on metrics after varying the number of features (Supplementary Figure S9 available online at http://bib.oxfordjournals.org/), which shows that using 60 features is enough to evaluate the approaches (Supplementary Figure S10 available online at http://bib.oxfordjournals.org/). For CATH, 10 features were selected by the distance between α carbons (the final list of features used can be found in Supplementary File 2 available online at http://bib.oxfordjournals.org/). A detailed description of feature selection procedures is available in Supplementary Materials available online at http://bib.oxfordjournals.org/.

### Preparation of training and test sets

After feature selection, class balancing was performed for both databases, using the last level as a reference. We tested six under-sampling methods [54–56] and two hybrid (over and under-sampling) methods [57, 58]. The best performance was observed with the Near Miss method, which is based on the nearest neighbors algorithm. The Near Miss heuristic rule selects the samples from the majority class that have the shortest average distance from the farthest samples of the negative class. Through the imbalanced-learn python toolbox [59], it was possible to make a semi-balance set containing more than 1000 samples on BioLip and 500 samples on CATH. Supplementary Figure S4 shows the class distributions before and after balancing approaches. For each level in Local approaches and for the last level in the Global approach, we used only classes that had at least 10 samples. This was employed to guarantee that one sample per fold would be available for cross-validation purposes. Therefore, the number of samples and classes may vary depending on the approach (Supplementary Files 1 and 2 available online at http://bib.oxfordjournals.org/).

**Figure 4:**
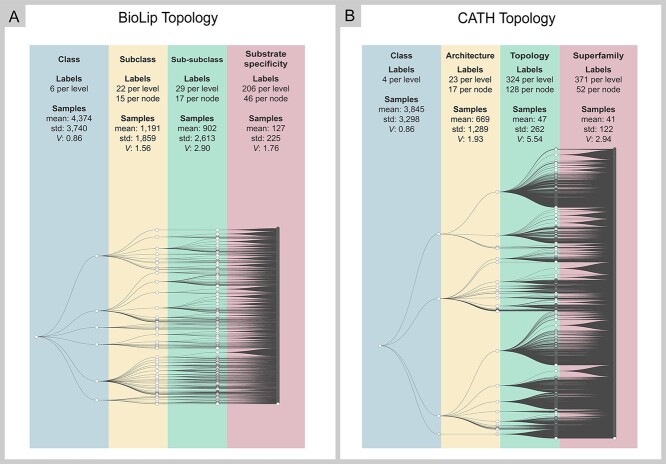
Topology of BioLip (**A**) and CATH (**B**) after class balancing and filtering. Each level shows the number of classes (labels) and the summary statistics of samples per class.

### Performance analysis of hierarchical approaches

We analyzed three hierarchical approaches: Global, Level and Node. In this regard, the datasets were tested and evaluated according to each approach. Initially, algorithm selection was performed with seven machine learning classifiers (Supplementary Table S4 available online at http://bib.oxfordjournals.org/). Three statistical learning classifiers were selected and compared: Decision Trees, Random Forest and Extremely Randomized Trees. Afterward, evaluations were performed by comparing the three approaches under 10-fold cross-validation [60, 61] and calculating Balanced Accuracy, MCC, AUC, *F*-score and Hierarchical measure [62].

As hierarchical methods require specific measures to evaluate results, we employed ‘hierarchical f-measure’ (hF), ‘hierarchical precision’ (hP) and ‘hierarchical recall’ (hR), originally proposed by Kiritchenko *et al*. [62] and recommended by Silla and Freitas [18, 62]. These measures consider not only the leaf prediction but also all ancestors of the class in a hierarchical graph, except for the root. Equations (1) and (2) depict hP and hR. These measures combined are presented in hF (Equation 3), in which $Ci$ and $Zi$correspond, respectively, to a set of test and predicted classes for an instance $i$.

(1) \begin{equation*} \mathrm{hP}=\frac{\varSigma i \mid Zi\cap Ci\mid }{\varSigma i \mid Zi\mid } \end{equation*}

(2) \begin{equation*} \mathrm{hR}=\frac{\varSigma i\mid Zi\cap Ci\mid }{\varSigma i\mid Ci\mid } \end{equation*}

(3) \begin{equation*} \mathrm{hF}=\frac{\ 2\ast \mathrm{hP}\ast \mathrm{hR}}{\mathrm{hP}+\mathrm{hR}} \end{equation*}

## Results

In this section, we evaluate Global and Local approaches in hierarchical datasets. Machine learning models were built following feature extraction and selection using three different algorithms (Decision Tree Classifier, Random Forest Classifier and Extra Trees Classifier) and assessed under 10-fold cross-validation.

The balance and filtering tasks applied in both data sets decreased the dispersion of the samples in the last level, considering class distribution per level and per node. In both data sets, we achieved a substantial decrease in the relation between the mean and the SD of the samples, represented by the Variation coefficient (*V*) in the last level (Figure 4).

### Local approaches: level-by-level behavior

**Figure 5:**
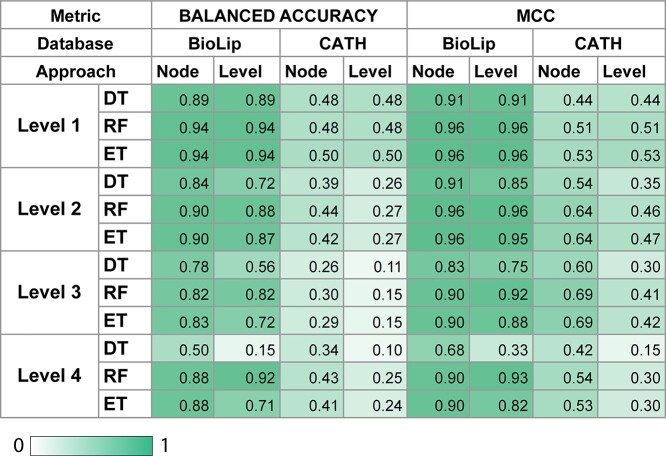
Metrics of model selection results by level for local approaches. Comparison of approaches in model selection between Decision Tree Classifier (DT), Random Forest Classifier (RF) and Extra Trees Classifier (ET) using Balanced Accuracy and MCC. Gradient colors indicate maximum results in dark green and minimum in white.

In this section, we analyze the results between Local approaches, which are divided into Node and Level. These approaches were compared using Balanced Accuracy and MCC metrics, time and memory used to train respective models in each level. Conventional performance metrics, AUC and *F*-score were also calculated (Supplementary Figure S5 available online at http://bib.oxfordjournals.org/). The results are summarized in Figure 5. In the next subsections, we describe and analyze the model behavior at each level.

#### Level 1

The effort to classify hierarchical datasets on the first level was the same in both approaches (Level and Node) since both generated only one model at this level. The difference between them is related to the number of predicted classes. In the BioLip dataset, the model predicted six classes; whereas in the CATH dataset, four classes were predicted (Figure 4). Class representation is unbalanced, and the number of labels for each class varies from 1 to 21 in CATH and from 6 to 22 in BioLip (Supplementary File 3 available online at http://bib.oxfordjournals.org/). As for samples, CATH has a large majority class that represents 52.54% of samples, followed by two classes of intermediate size (24.43% and 22.86% respectively), and a smaller class with 0.17% of the samples. In turn, BioLip has a better class distribution, with the three largest ones encompassing 34.11%, 32.2% and 19.91% of samples, respectively. The other three classes in BioLip have 8.33%, 2.87% and 2.55% of samples (Supplementary File 3 available online at http://bib.oxfordjournals.org/).

**Figure 6:**
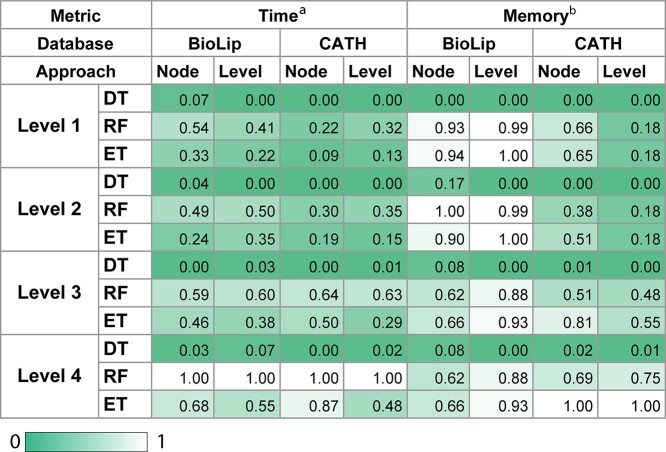
Time and memory measurement of model selection results. Comparison in model selection between Decision Tree Classifier (DT), Random Forest Classifier (RF) and Extra Trees Classifier (ET). Gradient colors indicate maximum results in dark green and minimum in white. ªTime in minutes. ^b^Memory in GB.

No difference (${P}_{\mathrm{value}}>0.23$, Student’s *t*-test) in predictive performance between the different models assessed within each data set was observed, with BioLip showing slightly better results than CATH (Figure 5). The running time required to train BioLip with Random Forest, however, was 63% higher than with Extra Trees, which presented a similar performance, based on Balanced Accuracy and AUC (Figure 6).

#### Level 2

On the second level of hierarchy, the characteristics of both datasets remained similar: Both produced a model with 22 and 23 classes in Level approach, respectively. In Node approach, BioLip had 15 classes per node and CATH had 17 (Figure 4). *V* is also close for both datasets; however, on this level, it exceeds 1, which means the data have a high variance. On this level, we observed a difference between performance scores for Level and Node approaches for both datasets (Figure 5), with Node outperforming Level in both (Figure 5). This difference was only observed for CATH.

#### Level 3

On the third level, differences (${P}_{\mathrm{value}}<0.01$, Student’s *t*-test) in the main characteristics of datasets may be responsible for the different behaviors observed on model selection tasks. On this level, the high dispersion of classes in CATH was evident, jumping from 23 classes per level to 324 and from 17 per node to 128. There is also a high variance in samples, with *V* values increasing almost 3-fold in comparison with the second level. The changes in BioLip were less significant. It had 22 classes per level on the second level and increased to 29 on the third. On the other hand, it had 17 classes per node on the second level, and this number remained the same on the third level.

CATH presented significant differences (${P}_{\mathrm{value}}<0.01$, Student’s *t*-test) between approaches, with better performance for the Node approach, despite higher training time. No significant difference was observed for BioLip. Extra Trees were more efficient in terms of running time than Random Forest for the Node approach, achieving similar predictive scores, with a slight difference in memory usage. Random Forest presented good results with the Level approach, including less memory usage than Extra Trees, and spending less time in comparison with the Node approach. At this level, both approaches have used memory similarly, an average of 5 GB for the Node approach and 6 GB for Level (Figure 6).

#### Level 4

Finally, on the last level, as we used a semi-balancing technique, there was an improvement in relation to the *V* values of samples compared with the third level. CATH consistently presented a higher *V* than BioLip. On this level, the same pattern was observed, that is, only CATH presented differences between approaches (Supplementary Table S1 available online at http://bib.oxfordjournals.org/).

When comparing Level and Node approaches, there was a significant difference in memory usage in the CATH. This might be associated with the number of classes being much higher in the Local approach by Level (371) than in the Local approach by Node (52) (Supplementary File 3 and Supplementary Table S6).

In short, when we compared Local approaches, there were no differences (${P}_{\mathrm{value}}>0.28$, Student’s *t*-test) (Supplementary Table S1 available online at http://bib.oxfordjournals.org/) between Node and Level for BioLip. For a database that has less class unbalance and partial depth labeling, the level approach could be a better option, considering the complexity of implementing the Node approach. In CATH, a database with full prediction depth and a high *V* in some levels, a significant difference (${P}_{\mathrm{value}}<0.01$, Student’s *t*-test) between the Level and Node approaches was observed. The Node approach produced more specific models, being a better option in this case. In the next section, we compare the Local approaches with the simplest way to classify hierarchies: the Global approach.

### Global versus Local approaches

**Figure 7:**
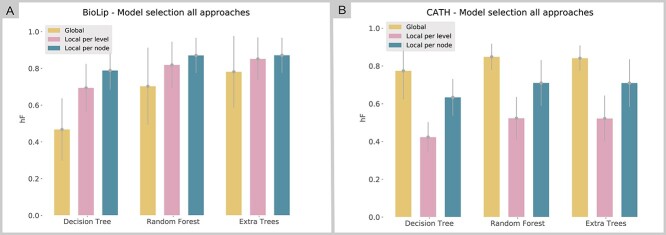
Comparison between Decision Trees, Random Forest and Extra Trees algorithms on model selection using the hierarchical metric (hF). (**A**) Model selection performed in the BioLip dataset comparing Global, Local per Level and Local per Node approaches. (**B**) Model selection performed in the CATH dataset comparing Global, Local per Level and Local per Node approaches. Error bars refer to the SD of performance for each algorithm.

To fairly compare the Global with Local approaches, we used hF measure considering the last level result of Local approaches. Figure 7 shows the mean hF measure under 10-fold cross-validation. In general, as expected, Extra Trees and Random Forest had better performance in both databases in all approaches. On the other hand, we observed a different behavior among the datasets toward the approaches. While CATH performed better using the Global approach, BioLip has a better result using Local approaches, as confirmed by previous analyses. In addition, there was no difference (${P}_{\mathrm{value}}>0.09$, Student’s *t*-test) between the Global and both local approaches for BioLip, while this difference is significant for CATH (Supplementary Table S2 available online at http://bib.oxfordjournals.org/).

**Table 2:** Comparison between classes used to train models in Global approach × Local approaches

	Global		Level (last level)		Node	
	CATH	BioLip	CATH	BioLip	CATH	BioLip
Classes	204	589	654	206	608^a^	672^a^
Samples/class	40	15	47	127	29	68
SD^b^	61	35	510	225	980	252

^a^Calculated from the mean number of classes multiplied by number of models.

^b^SD of number of samples per class.

Better results using the Global approach for CATH could be related to full depth labeling. In the Global approach, we may consider only the samples classified until the last level. Another factor that may have contributed to this observed result is the lack of information flow in CATH. CATH topology does not follow an evolutive path, like other biological datasets (e.g. Pfam [63], taxonomic databases [3, 25, 64–66], etc.); therefore, the use of Local approaches is not efficient in this context, as the hierarchy does not necessarily reflect evolutionary relationships from one level to another in the same branch, nor for nodes at the same level. Since the fourth level of CATH is different from the previous ones (and does not have strict classification criteria), we also performed an assessment just using the first three levels of the hierarchy (Supplementary Figure S6 available online at http://bib.oxfordjournals.org/). Interestingly, the results obtained at level 3 were consistent with those using the full hierarchy.

For BioLip, as expected, a considerably larger number of classes in the Global approach led to worse performance in comparison with local models (Table 2). Furthermore, Local approaches are more specific. This allows multiple models that can handle more classes overall in comparison with the Global approach to also handle partial depth classes.

**Figure 8:**
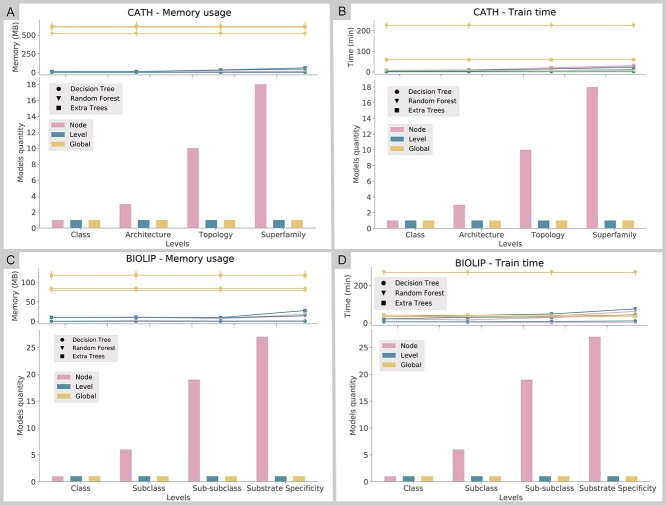
Number of models generated by Local and Global approaches related to time and memory spent on training tasks. The *y* axis was divided into two parts: at the top, we represent the Memory usage, for **A** and **C**, and Training time, for **B** and **D**; at the bottom, we represent Models quantities in all the panels. (**A**) Memory usage of CATH. (**B**) The training time of CATH. (**C**) Memory usage of BioLip. (**D**) The training time of BioLip. Error bars refer to the SD of the time and memory means for each algorithm.

Figure 8 shows the memory usage and processing time comparison between approaches, as well as the difference in the number of models for each Local approach on each level. The Level approach produced one model per level, with intermediate memory usage and processing times on each level. The Global approach produced one model gathering all levels, with constant memory usage and processing time regardless of the level. As the number of classes grew and depth increased, more time and memory were necessary to train the models.

The Node approach produced one model for each parent node in the hierarchy. The models were simpler than those in the Level approach; however, it was necessary to deal with a larger number of models. We can observe that, in comparison with the Global approach, the Local approach consumed less memory (Figure 8A and C), as it handled fewer data per model. However, the training time varied drastically depending on the algorithm used (Figure 8B and D). Random Forest, for both CATH and BioLip, presented the highest training times, with Decision Trees being the most efficient algorithm.

As expected, the Global approach was the most computationally demanding due to the number of classes in a single model. Compared to the Local approaches per node and level, Global had 4012 classes for the CATH and 1692 for BioLip, while the local ones per node and level had a total of 654 and 206 classes, respectively (Supplementary File 3 available online at http://bib.oxfordjournals.org/).

Nonetheless, when the database has full depth labeling and the classification goal is related to sensibility, performing the experiments with the Global approach may be an adequate and interesting alternative. Otherwise, even if the database has full depth labeling, if the classification goal involves specificity, it might be advantageous to consider Local approaches to achieve a more running time-efficient classification.

Alternatively, when we have partial depth labeling other components must be considered, including class dispersion and the computational resources available. According to our results, if the database has low dispersion in the levels or the computational resources are limited, the Level approach is more suitable. The Node approach tends to be appropriate in situations of high dispersion of data and when time and computing resources are not a restraint.

### Guidelines for modeling hierarchical classification

**Figure 9:**
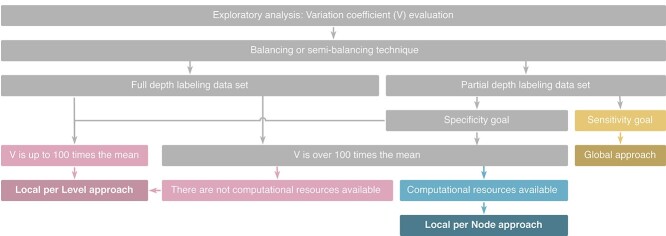
Guidelines to perform a hierarchical analysis. The workflow uses the Variation coefficient (*V*) (used to measure the variation of data); the challenges usually faced in hierarchical datasets, such as unbalanced samples, and prediction by depth (divided into partial depth labeling and full depth labeling); and the availability of computational resources to guide the choice of an appropriated classification approach: Global, Local per Level or Local per Node.

Based on the results discussed above, we developed an initial guideline to help the decision-making process of modeling hierarchical classification problems for biological data sets. The flowchart in Figure 9 describes the choice of approaches to use, considering the classification challenges detected on the data set. The components of classification challenges we considered are depth level classification, prediction by the depth and unbalanced classes.

Starting from an exploratory analysis, we recommend analyzing the unbalanced nature of classes at the last level, using the Variation coefficient (*V*), which indicates the dispersion of samples related to its mean. In unbalanced databases, the ideal approach is to apply a balancing or semi-balancing technique to the data. The semi-balancing technique is preferably used when classes have a limited number of samples, to avoid the sub-sampling of classes, which makes model generalization difficult.

If the database has partial depth labeling, we suggest adopting one of the Local approaches, using *V* to guide the decision between Local per Level and Local per Node approaches. When *V* is up to 100 times the mean ($V\le 1$) [34], the Local per Level approach is enough, achieving good performance while using fewer computational resources. On the other hand, samples with $V>1$ are considered highly dispersed, thus consuming more computational resources. In this case, we advise performing the classification using Local per Level only when there are not enough computational resources available; otherwise, we suggest using the Local per Node approach.

For databases that present full-depth labeling, the criteria that should guide the next steps is the goal of prediction. Evaluating Machine Learning models in terms of sensitivity and specificity can be described as the capacity of the predictor to detect true positives and true negatives, respectively. When the predictive modeling goal involves sensitivity, adopting a Global approach is adequate, despite being more computationally costly. This is also true when the database has partial depth labeling. Alternatively, if the database has full-depth labeling, and the classification goal involves specificity, it is necessary to consider Local approaches to achieve better classification performance.

While the goal of these broad guidelines is not to restrict the modeling process (e.g. an empirical assessment is still required), these suggestions could be used as initial guidelines for the analysis of hierarchical datasets up to four levels. It is essential to start with a detailed exploratory analysis of the dataset to identify which hierarchical classification challenges are to be overcome.

As a suggestion for using our guideline for future analyses, we make some recommendations based on the characteristics of the databases, which could be consistent for databases with similar characteristics (Supplementary Figures S7 and S8 available online at http://bib.oxfordjournals.org/). For CATH, if the goal of the work is specificity, we suggest Local approaches be prioritized. If the main goal is specificity, a Global approach might be more adequate. Similar lessons can be potentially applied to other databases with the same structure and domain, such as Pfam (Supplementary Table S3 available online at http://bib.oxfordjournals.org/). For BioLip, we suggest one of the Local approaches: if few computational resources are available, the Level approach may be the best option; otherwise, the Node approach is an interesting option. Additionally, since KEGG also has similar challenges and structures to BioLip, the same lessons could be potentially applied to it. Looking at challenges and structure from the other databases we reviewed (Supplementary Table S3 available online at http://bib.oxfordjournals.org/) and applying our guideline, we suggest using the Local approach for Silva, GreenGenes, RDP, OTT and NCBI Taxonomic. We hope the community extends this analysis to these databases in the future. Supplementary Figure S8 summarizes these suggestions.

## Conclusions

The level approach produced a single model to classify each level, rather than a single, large model as the Global approach. The Node approach produced a model for each node on each level of the hierarchy, producing more specific models, consequently using less memory for each model. Surprisingly, the Global approach presented better results than the Local approaches for the CATH database, which we hypothesize could be linked to one of the evaluated components of hierarchical challenges, the prediction by depth.

Considering the analysis of samples per class is also important to further refine the decision-making process between the approaches and the number of classes per model. In future works, we intend to provide computational libraries to help the community in the decision process to model hierarchical data.

In this work, we provided a guideline to support the decision-making process toward an approach to achieving more robust and generalizable models to classify hierarchical data. This guideline is an initial proposal toward rationalizing hierarchical classification strategy prioritization based on data set properties. We hope to provide initial evidence to support further discussion within the scientific community, which can lead to further assessment on different biological scenarios. While this work primarily focused on biological data, we believe this guide could be applied to other domains of knowledge where hierarchical data are available.

**Key Points**

- Many biological data sets are hierarchical in nature and these hierarchies become a challenge for classification tasks, once hierarchical classification associate labels to different classification levels.
- Previous efforts have shown that classifiers tailored to this type of complex hierarchical data can improve information retrieval effectively and describe the main challenges in hierarchical classification tasks; however, little has been done to comprehensively assess the utility, applicability and limitations of different hierarchical classification approaches.
- We evaluated the approaches previously proposed (Global, Local per Level and Local per Node) and applied them to two different biological databases (CATH and BioLip) to investigate their pros and cons and establish general guidelines of practice.
- We showed how different components of hierarchical data sets can guide the decision process between the approaches.
- The guidelines provided in this work could support the hierarchical classification tasks, which could potentially optimize computational resources and performance.

## Supplementary Material

Supplementary_file_1_bbac216

Supplementary_file_2_bbac216

Supplementary_file_3_bbac216

Supplementary_Material_R2_bbac216

certificate_english_revision_bbac216

## Data Availability

The data sets used in the study are available as supplementary materials.

## Author Biographies

**Pâmela M. Rezende** is a Data Science Leader at Stilingue and Ph.D. candidate in Bioinformatics at the Federal University of Minas Gerais, collaborating with the René Rachou Institute and the University of Melbourne.

**Joicymara S. Xavier** is Assistant Professor of Computer Science at the Federal University of Vales do Jequitinhonha and Mucuri and Ph.D. candidate at Universidade Federal de Minas Gerais, collaborating with the René Rachou Institute and the University of Melbourne.

**David Ascher** is Deputy Director of Biotechnology at The University of Queensland, and head of Computational Biology and Clinical Informatics at the Baker Institute and Systems and computational Biology at Bio21 Institute. He is interested in developing and applying computational tools to assist leveraging clinical and omics data for drug discovery and personalised medicine.

**Gabriel R. Fernandes** is a researcher at Rene Rachou Institute - Fiocruz Minas, and an associate researcher at the Center for Basic Metabolic Research at the University of Copenhagen-Denmark. He specialises in Bioinformatics, Genomics and microbiome analysis.

**Douglas E. V. Pires** is a Senior Lecturer in Digital Health with the School of Computing and Information Systems at the University of Melbourne and group leader at Bio21 Institute. He is a computer scientist and bioinformatician specialising in machine learning and AI and the development of tools to analyse omics data.
