# Supplementary material for: Evaluating hierarchical machine learning approaches to classify biological databases
Source: Brief Bioinform. 2022 Jun 21;23(4):bbac216. doi: 10.1093/bib/bbac216 (PMC9310517; doi:10.1093/bib/bbac216)
Supplement: Supplementary_Material_R2_bbac216 [file supplementary_material_r2_bbac216.pdf]

SUPPLEMENTARY MATERIALS

Evaluating Hierarchical Machine Learning Approaches to Classify Biological Databases

Pâmela M. Rezende[1,2,3],Joicymara S. Xavier[1,2,4], David B. Ascher[5,6,7*],
Gabriel R. Fernandes[2*], Douglas E. V. Pires[6,7,8*]

[1]Universidade Federal de Minas Gerais;
[2]Instituto René Rachou, Fundação Oswaldo Cruz;
[3]Stilingue Inteligência Artificial;
[4]Institute of Agricultural Sciences, Universidade Federal dos Vales do Jequitinhonha e Mucuri;
[5]School of Chemistry and Molecular Biosciences, University of Queensland
[6]Systems and Computational Biology, Bio 21 Institute, University of Melbourne;
[7]Computational Biology and Clinical Informatics, Baker Heart and Diabetes Institute;
[8]School of Computing and Information Systems, University of Melbourne

*To whom correspondence should be addressed. Tel: +61 3 8344 8185; Email: douglas.pires@unimelb.edu.au. Correspondence may also be addressed to d.ascher@uq.edu.au and gabriel.fernandes@fiocruz.br.

FIGURES

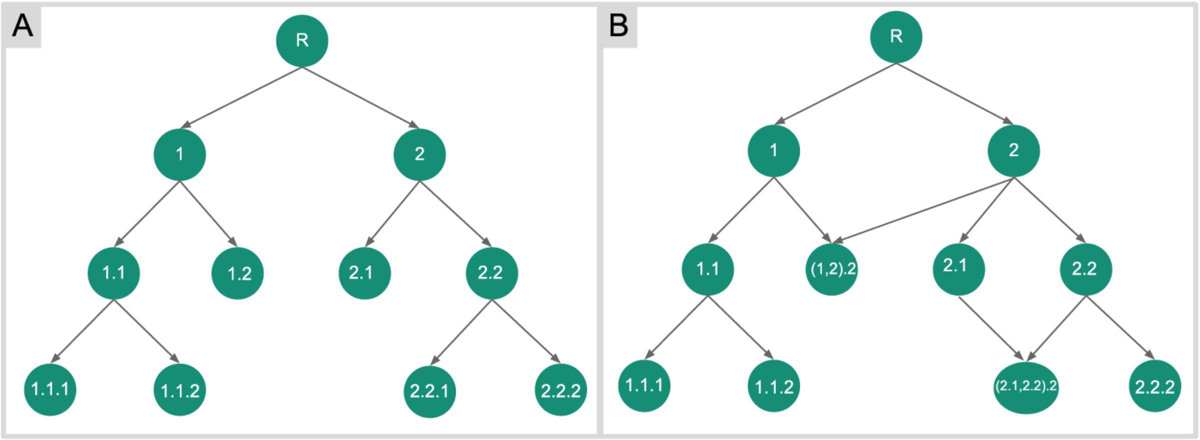

Figure S1: Hierarchy examples of the difference between Tree and DAG topologies.: (A) Tree: an undirected graph in which any edges are connected by exactly one node and (B) Directed Acyclic Graph (DAG): A direct graph with no directed cycles. The edges of DAG only go one way, nonetheless, a node could be connected by multiple edges.

Local analysis was performed on an Intel(R) Xeon(R) CPU E7- 4850 @ 2.00GHz, 80 cores, 65.78 GB of memory, CentOS Linux 7 (Core)
Global analysis was performed on an Intel(R) Xeon(R) CPU E5-2670 v3 @ 2.30GHz, 48 cores, 395.984 GB of memory, CentOS Linux 7 (Core).

Figure S2: Configuration of machines used to perform the experiments.

CATH: version 4.2
BioLip: version 2019-07-15
Silva: version 128
KEGG: version 2022-03-10

Figure S3: Used versions of data sets.

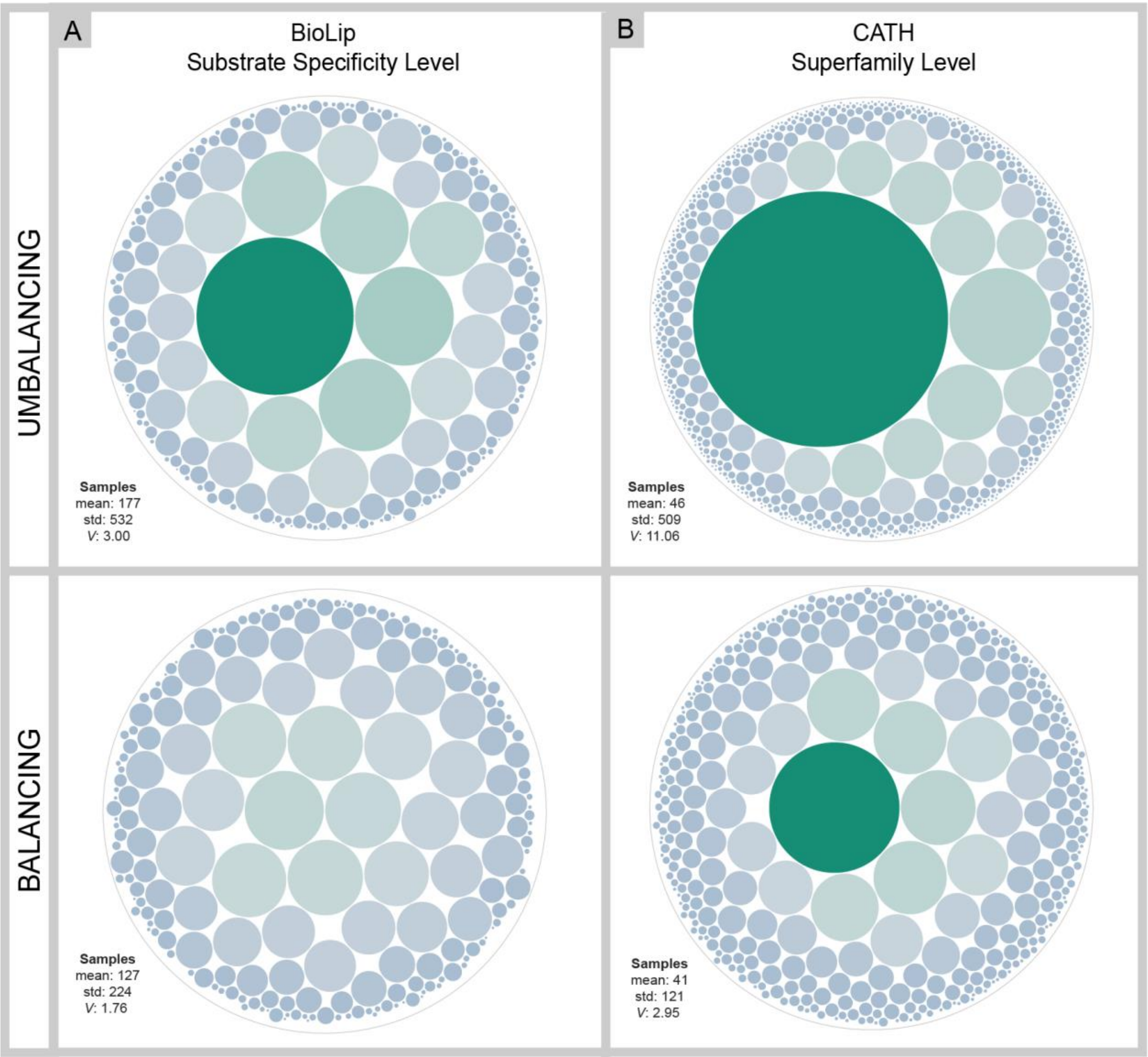

Figure S4: Statistics of samples before and after balancing each database. Variation coefficient (*V*) represents the ratio between the standard deviation (STD) and the mean.

Metric		AUC				F-SCORE			
Database		BioLip		CATH		BioLip		CATH	
Approach		Node	Level	Node	Level	Node	Level	Node	Level
Level 1	DT	0.95	0.95	0.76	0.76	0.94	0.94	0.66	0.66
	RF	0.98	0.98	0.80	0.80	0.97	0.97	0.71	0.71
	ET	0.98	0.98	0.81	0.81	0.97	0.97	0.72	0.72
Level 2	DT	0.96	0.93	0.80	0.72	0.94	0.87	0.63	0.48
	RF	0.98	0.97	0.84	0.77	0.97	0.96	0.72	0.57
	ET	0.98	0.97	0.84	0.77	0.97	0.96	0.71	0.59
Level 3	DT	0.93	0.91	0.82	0.68	0.88	0.83	0.64	0.37
	RF	0.96	0.97	0.86	0.74	0.93	0.95	0.73	0.50
	ET	0.96	0.95	0.86	0.74	0.93	0.92	0.73	0.50
Level 4	DT	0.93	0.91	0.71	0.58	0.69	0.29	0.44	0.18
	RF	0.96	0.97	0.77	0.65	0.91	0.93	0.55	0.33
	ET	0.96	0.95	0.76	0.65	0.91	0.82	0.54	0.33

0 1

Figure S5: Metrics of model selection results by level for local approaches. Comparison of approaches in model selection between Decision Tree Classifier (DT), Random Forest Classifier (RF), and Extra Trees Classifier (ET) using AUC and F-score metrics. The values used refer to the last fold of 10-fold cross-validation. Heatmap colors indicate maximum results in dark green, minimum in shades of red, and midpoint in gray.

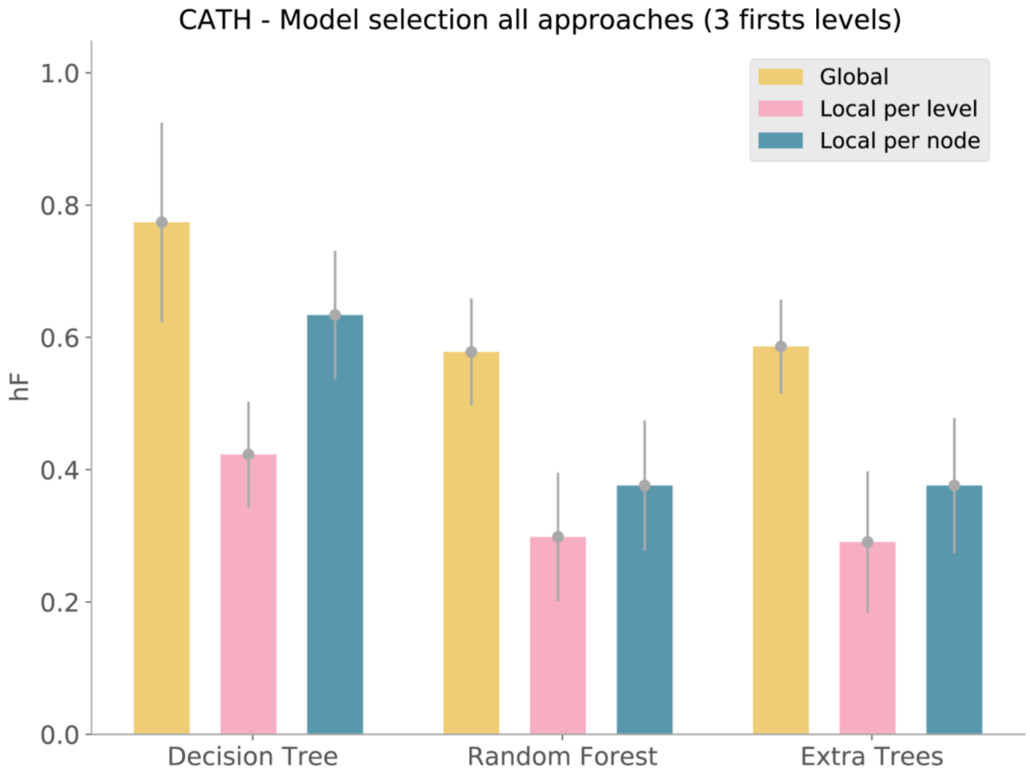

Figure S6: Comparison between Decision Trees, Random Forest, and Extra Trees algorithms on model selection using the hierarchical metric (*hF*). Model selection performed, we used 3 first levels in the CATH dataset comparing Global, Local per level, and Local per node approaches. Error bars refer to the standard deviation of the time and memory for each algorithm.

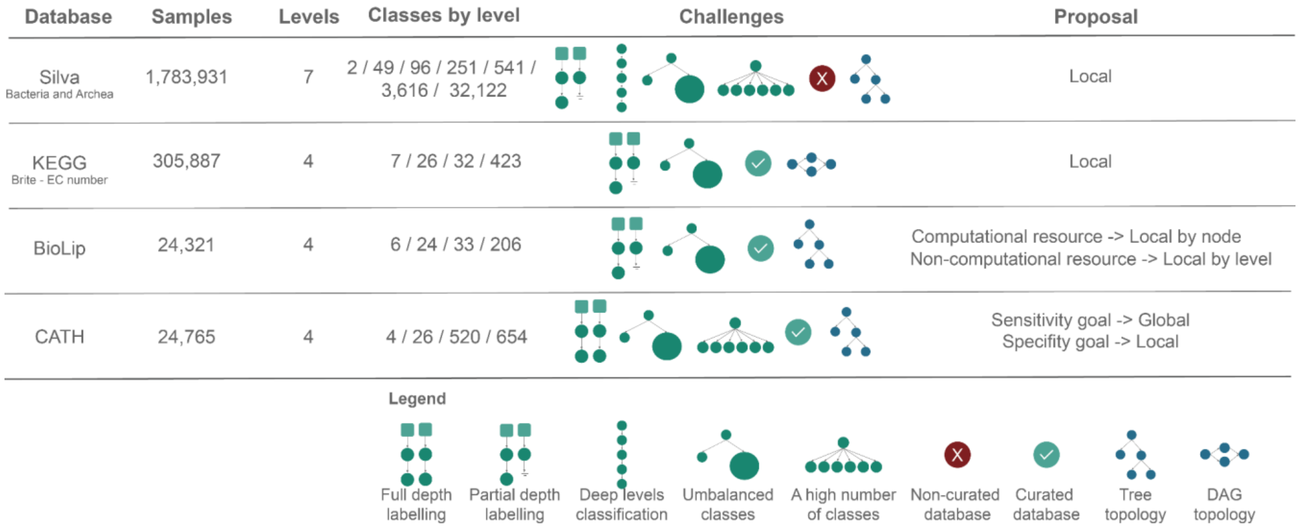

Figure S7: Exploratory analyses for different hierarchical biological datasets. The analyses were performed using Silva (Bacteria and Archea), KEGG (Brite - EC number), BioLip, and CATH in order to understand the main characteristics of hierarchical biological datasets from different domains. We evaluate statistical numbers for each dataset (number of samples (Samples), number of levels in the hierarchy (Levels), and number of classes present in each level (Classes by Level), Challenges, and the Proposal. Challenges refer to the problems faced by hierarchical classification, database curation, and topology. The problems faced by hierarchical classification usually are: Prediction by deep (Full-depth labeling and Partial depth labeling), Deep levels classification, Unbalanced classes, and a High number of classes. Relating to database curation, we used a binary classification: Non-curated or curated, and for the topology, the datasets follow a Tree or DAG (Directed Acyclic Graph). In the Proposal column, we suggested approaches that can be used for each database applying our guidelines. Note that for Silva and KEGG we are generalizing the results from the experiments performed in this work, based on an exploratory analysis accomplished using both datasets.

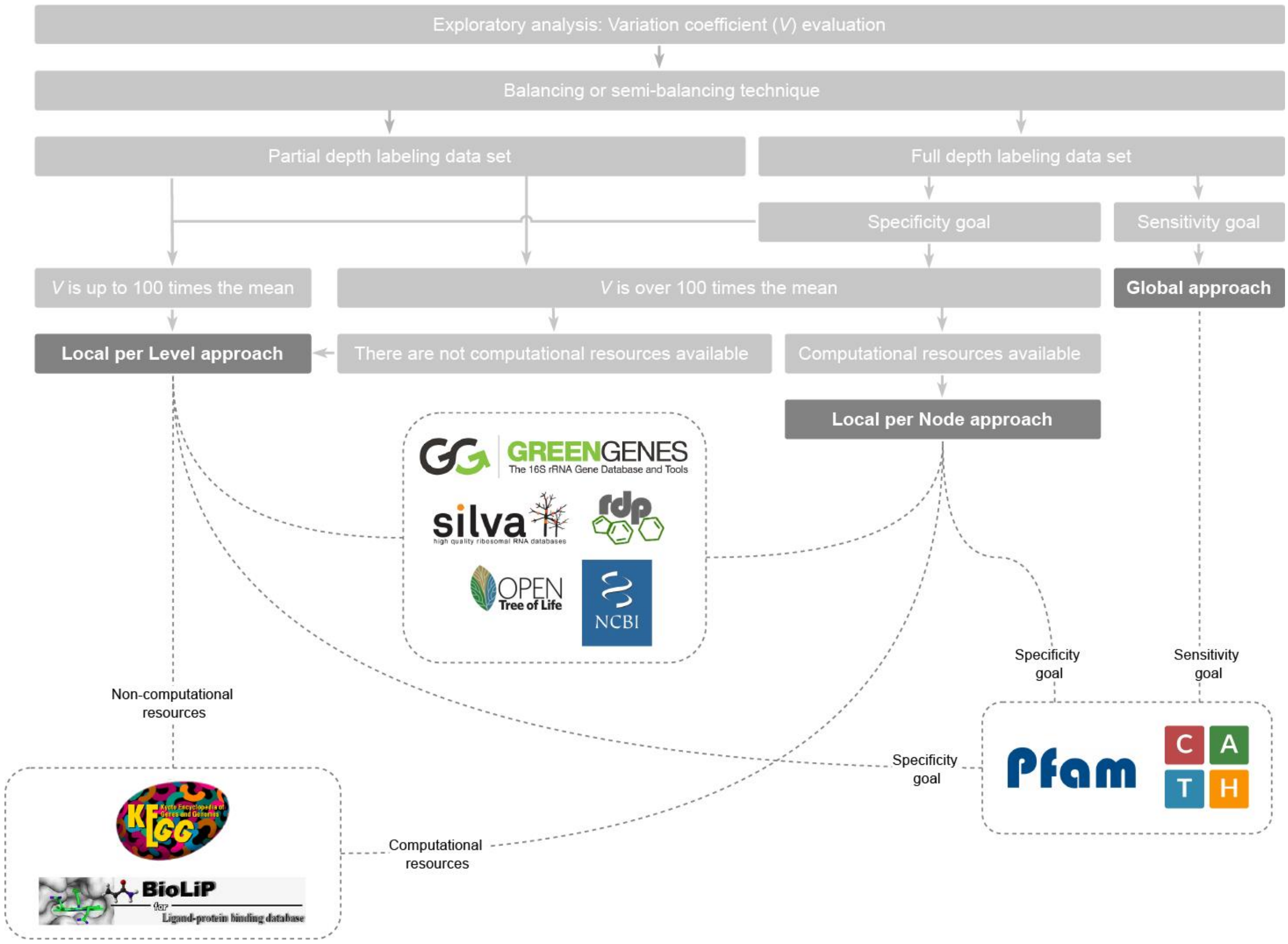

Figure S8. Summary of extending hierarchical learning approaches to different databases.

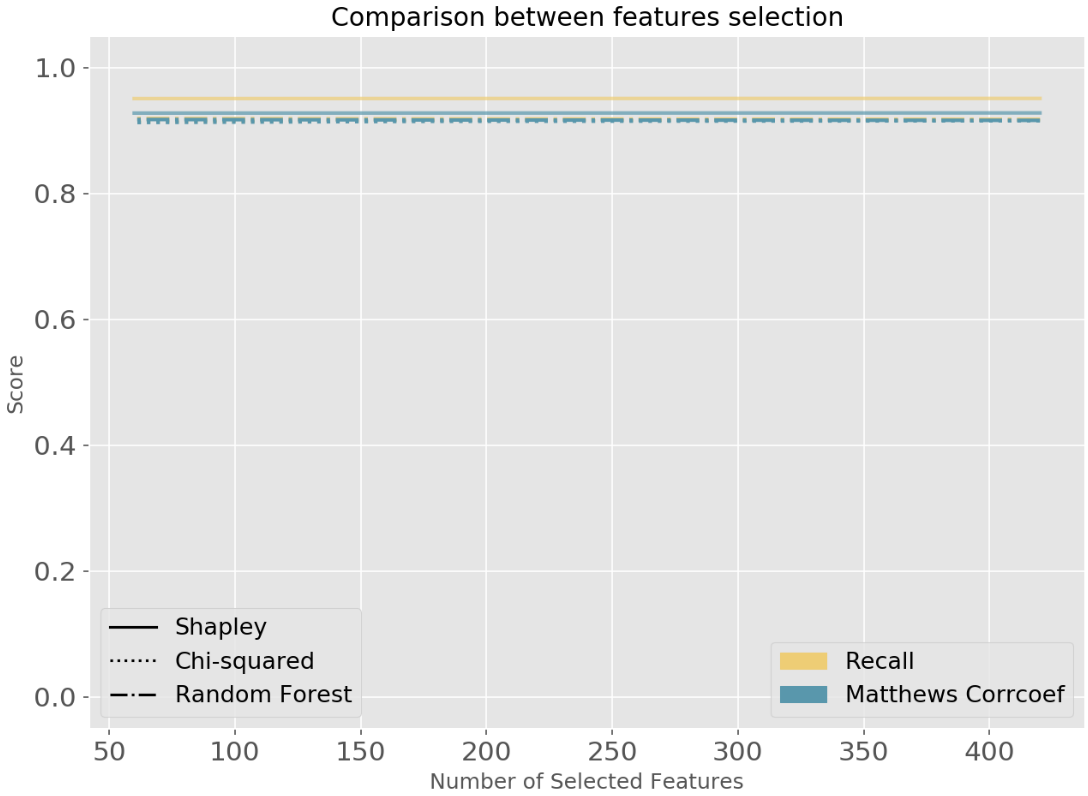

Figure S9. MCC and recall for comparison between Shapley value, Chi-squared, and importance of traits methods by Random Forest method for trait selection, varying the number of traits in BioLip.

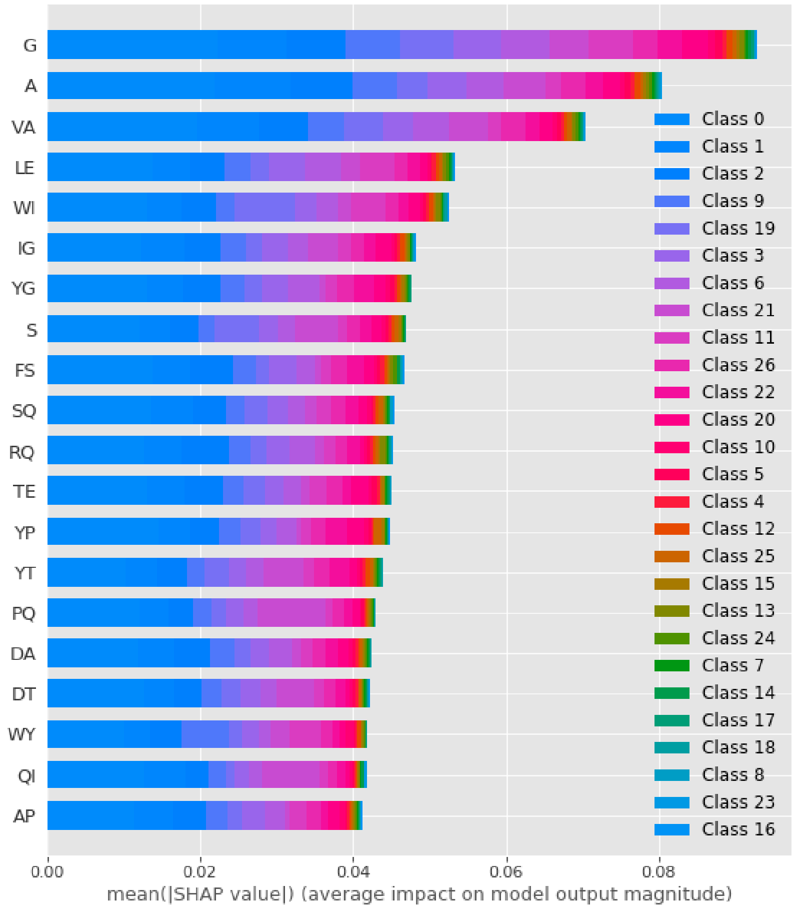

Figure S10. Figure automatic generate by Shapley value with the first 20 most important features for BioLip. Since we noticed that from 60 features, the number of them did not change the results, we selected the 60 most important features, according to Shapley.

TABLES

Table S1: Statistical student's t-tests to evaluate differences between Level and Node approaches

			**p-value**
**BL ACC**	**Biolip**	**Level 2**	0.2278
	**Biolip**	**Level 3**	0.2188
	**Biolip**	**Level 4**	0.2860
**BL ACC**	**CATH**	**Level 2**	0.0049
	**CATH**	**Level 3**	0.0009
	**CATH**	**Level 4**	0.0100
**MCC**	**Biolip**	**Level 2**	0.3788
	**Biolip**	**Level 3**	0.4567
	**Biolip**	**Level 4**	0.3526
**MCC**	**CATH**	**Level 2**	0.0019
	**CATH**	**Level 3**	0.0008
	**CATH**	**Level 4**	0.0031

Table S2: Statistical student's t-tests to evaluate differences between Global and Local approaches.

			**p-value**
		**Node - Global**	0.1046
	**Biolip**	**Level - Global**	0.0977
		**Node - Global**	0.0004
**hF**	**CATH**	**Level - Global**	0.0009

Table S3: Review of most used publicly available biological hierarchical databases

Database	Domain	Citations*
BioLip [1]	Protein function classification	463
CATH [2–5]	Protein structure classification	3128
Pfam [6]	Protein domain classification	2877
GreenGenes [7]	Taxonomic classification	9759
KEGG Brite - EC Number [8]	Protein function classification	4791
NCBI taxonomy [9]	Taxonomic classification	1072
OTT [10]	Taxonomic classification	523
RDP [11]	Taxonomic classification	3265
Silva [12]	Taxonomic classification	1651

* Citations on Google Scholar in March 2022.

Table S4: Model selection with hierarchical databases (CATH, BioLip and Silva) using 10 folds in cross-validation.

Models	Best fold accuracy
XGBoost	0.60
Random Forest	0.86
Decision Tree	0.79
Extra Trees	0.78
Radius Neighbors	0.08
Ridge	0.49

Table S5: Statistical student's t-tests to evaluate differences between BioLip and CATH levels.

		**p-value**
**Time - Node**	**Level 2**	0.2020
	**Level 3**	0.1668
	**Level 4**	0.5212
**Time - Level**	**Level 2**	0.1926
	**Level 3**	0.4993
	**Level 4**	0.1926
**Memory - Node**	**Level 2**	0.0935
	**Level 3**	0.9226
	**Level 4**	0.4252
**Memory - Level**	**Level 2**	0.1826
	**Level 3**	0.1882
	**Level 4**	0.7707

Table S6: Statistical student's t-tests to evaluate differences between Level and Node approaches

		**p-value**
**Time**	**Biolip**	0.1751
**Time**	**CATH**	0.3657
**Memory**	**Biolip**	0.1081
**Memory**	**CATH**	0.0256

SUPPLEMENTARY METHODS

A. Data set preparation

CATH's dataset preparation

We downloaded from the CATH website[1], all protein domains with available structural data in the Protein Data Bank (PDB) with their respective classifications. Features were generated based on the CSM [13] algorithm.

BioLip's dataset preparation

Enzyme Classification numbers (EC numbers) available on the BioLip website[2] were retrieved and FASTA files with the protein sequences, downloaded and presented to iFeatures [14] for feature generation.

B. Feature Engineer and Selection

We used different features sets for each dataset, based on previous well established approaches that employed each dataset. We used amino acid composition descriptors from iFeature [14] to represent proteins in Biolip [1] as this set of features is broadly used for other works in the related field [15–17] and graph-based signatures [13] to represent protein structures in CATH, which have been previously used to model protein structure and function [17–19] and predict effects of mutation [20–22].

Feature engineer on CATH

Cutoff Scanning Matrix (CSM) [13] generates feature vectors that represent distance patterns between protein residues. The motivation behind CSM is the fact that proteins with different folds and functions have significantly different distributions of distances between their residues, and protein similarity is reflected in these distance distributions. CSM generates signatures based on the minimum and maximum distances between alpha carbons in a structure [13]. This distance is scanned with an incremental step.

We generated 100 sets of features using 5Å as minimum and 30Å as maximum distance with a step of 2Å. In order to choose only one feature set, we performed 10-fold cross-validation with three different algorithms, Decision Tree, Extra Tree, and Random Forest, with the best set containing 10 features in total (Supplementary file 2).

---

[1] https://www.cathdb.info/download
[2] https://zhanggroup.org/BioLiP/library.html

Feature selection and engineering on BioLip

iFeature [14] is a Python toolkit for calculating a wide range of structural and physicochemical feature descriptors from proteins and peptide sequences. We extracted 420 features using the different descriptors available on iFeature. In order to select the most important features to describe BioLip data, we performed a 10-fold cross-validation and evaluated MCC by varying the number of selected features, based on the Grid search technique [23]. After each grid search step, we analyzed feature importance using the Shapley value [24]. We have compared Shapley value results with two other feature selection technics: Chi-squared and Random Forest Importance (Figure S9). Using this comparison we concluded that Shapley value is good in representing the dataset, instead of other methods. Therefore, we selected the 60 most important features, according to Shapley (Figure S10).

SUPPLEMENTARY REFERENCES

1. Yang J, Roy A, Zhang Y. BioLiP: a semi-manually curated database for biologically relevant ligand-protein interactions. Nucleic Acids Res. 2013; 41:D1096-103

2. Pearl FMG, Bennett CF, Bray JE, et al. The CATH database: an extended protein family resource for structural and functional genomics. Nucleic Acids Res. 2003; 31:452–455

3. Dawson NL, Lewis TE, Das S, et al. CATH: an expanded resource to predict protein function through structure and sequence. Nucleic Acids Res. 2017; 45:D289–D295

4. Orengo CA, Michie AD, Jones S, et al. CATH--a hierarchic classification of protein domain structures. Structure 1997; 5:1093–1108

5. Das S, Scholes HM, Sen N, et al. CATH functional families predict functional sites in proteins. Bioinformatics 2021; 37:1099–1106

6. El-Gebali S, Mistry J, Bateman A, et al. The Pfam protein families database in 2019. Nucleic Acids Res. 2019; 47:D427–D432

7. DeSantis TZ, Hugenholtz P, Larsen N, et al. Greengenes, a chimera-checked 16S rRNA gene database and workbench compatible with ARB. Appl. Environ. Microbiol. 2006; 72:5069–5072

8. Kanehisa M, Furumichi M, Tanabe M, et al. KEGG: new perspectives on genomes, pathways, diseases and drugs. Nucleic Acids Res. 2017; 45:D353–D361

9. Federhen S. The NCBI Taxonomy database. Nucleic Acids Res. 2012; 40:D136-43

10. Hinchliff CE, Smith SA, Allman JF, et al. Synthesis of phylogeny and taxonomy into a comprehensive tree of life. Proc Natl Acad Sci USA 2015; 112:12764–12769

11. Cole JR, Wang Q, Fish JA, et al. Ribosomal Database Project: data and tools for high throughput rRNA analysis. Nucleic Acids Res. 2014; 42:D633-42

12. Yilmaz P, Parfrey LW, Yarza P, et al. The SILVA and “All-species Living Tree Project (LTP)” taxonomic frameworks. Nucleic Acids Res. 2014; 42:D643-8

13. Pires DEV, de Melo-Minardi RC, dos Santos MA, et al. Cutoff Scanning Matrix (CSM): structural

classification and function prediction by protein inter-residue distance patterns. BMC Genomics 2011; 12 Suppl 4:S12

14. Chen Z, Zhao P, Li F, et al. iFeature: a Python package and web server for features extraction and selection from protein and peptide sequences. Bioinformatics 2018; 34:2499–2502

15. Shen Y, Tang J, Guo F. Identification of protein subcellular localization via integrating evolutionary and physicochemical information into Chou's general PseAAC. J. Theor. Biol. 2019; 462:230–239

16. Akcesme B. Prediction of Protein Structural Classes for Low-Similarity Sequences Based On Predicted Secondary Structure. scjournal 2015; 4:

17. Song J, Wang Y, Li F, et al. iProt-Sub: a comprehensive package for accurately mapping and predicting protease-specific substrates and cleavage sites. Brief. Bioinformatics 2019; 20:638–658

18. da Silva BM, Myung Y, Ascher DB, et al. epitope3D: a machine learning method for conformational B-cell epitope prediction. Brief. Bioinformatics 2022; 23:

19. da Silveira CH, Pires DEV, Minardi RC, et al. Protein cutoff scanning: A comparative analysis of cutoff dependent and cutoff free methods for prospecting contacts in proteins. Proteins: Structure, Function, and Bioinformatics 2009; 74:727–743

20. Pires DEV, Ascher DB, Blundell TL. mCSM: predicting the effects of mutations in proteins using graph-based signatures. Bioinformatics 2014; 30:335–342

21. Pires DEV, Ascher DB, Blundell TL. DUET: a server for predicting effects of mutations on protein stability using an integrated computational approach. Nucleic Acids Res. 2014; 42:W314-9

22. Pires DEV, Rodrigues CHM, Ascher DB. mCSM-membrane: predicting the effects of mutations on transmembrane proteins. Nucleic Acids Res. 2020; 48:W147–W153

23. Bergstra J, Bengio Y. Random Search for Hyper-Parameter Optimization. JMLR 2012; 13:281–305

24. Shapley LS. A value for n-person games. Contributions to the Theory of Games 1953; 2:307–317
