# Supplementary material for: Evaluating hierarchical machine learning approaches to classify biological databases
Source: Brief Bioinform. 2022 Jun 21;23(4):bbac216. doi: 10.1093/bib/bbac216 (PMC9310517; doi:10.1093/bib/bbac216)
Supplement: certificate_english_revision_bbac216 [file certificate_english_revision_bbac216.pdf]

DECLARATION

I, **Ernani Augusto de Souza Junior**, co-founder and English teacher at OnPoint English for Specific Purposes, and undergraduate student in Language Studies (Portuguese-English) from Universidade Federal de Lavras, hereby declare that I have revised the paper entitled "**Evaluating Hierarchical Machine Learning Approaches to Classify Biological Databases"** written by Pâmela M. Rezende, Joicymara S. Xavier, David B. Ascher, Gabriel R. Fernandes, and Douglas E. V. Pires. .

Thus, for all intents and purposes, and request of the interested party, I sign this letter to demonstrate the truthfulness of this statement.

Lavras, April 27th, 2022

Ernani Augusto de Souza Junior

Ernani Augusto de Souza Junior

OnPoint English for Specific Purposes
